# Usefulness of an educational lecture focusing on improvement in public awareness of and attitudes toward depression and its treatments

**DOI:** 10.1186/s12913-017-2071-0

**Published:** 2017-02-10

**Authors:** Takashi Yakushi, Teizo Kuba, Yuzuru Nakamoto, Hiroshi Fukuhara, Munenaga Koda, Osamu Tanaka, Tsuyoshi Kondo

**Affiliations:** 1 Department of Neuropsychiatry, Graduate School of Medicine, University of the Ryukyus, 207 Uehara, Nishihara-cho, Okinawa 903-0215 Japan; 2 Aomori Prefectural Center for Mental Health and Welfare, 353-92 Sannai Sawabe, Aomori, 038-0031 Japan

**Keywords:** Stigma, General population, Awareness, Attitudes, Depression, Suicide prevention, Anti-stigma-targeted lecture, Medical model, Help-seeking

## Abstract

**Background:**

There is an urgent need to establish effective strategies for suicide prevention. Stigma against depression may be a potential anti-protective factor for suicide. Thus, we investigated baseline levels of awareness and attitudes toward depression and its treatment among the general population by our original 18-item questionnaire, which we aimed to validate in the present study. Next, we conducted two types of educational interventions and examined the results to clarify the difference in the quality of these lectures.

**Methods:**

Subjects were 834 citizens (245 males and 589 females) who received an anti-stigma-targeted (*n* = 467) or non-targeted lecture (*n* = 367). An 18-item questionnaire assessing levels of awareness and attitudes toward depression and its treatments was administered to each participant before and after the lecture. A chi-square test was used to investigate categorical variables for background data on the participants. Factor analysis of baseline scores was conducted on the 18 questionnaire items. Student’s *t*-test was used for analysis of the gender effect. A two-way analysis of variance (ANOVA) was used for comparison among the 5 age groups and comparison of the effect of the two lectures. Multiple regression analysis was applied to examine the determinants of improved attitudes after intervention.

**Results:**

Public attitudes toward depression consisted of 4 distinct elements, which were disease-model attitudes, help-seeking behavior, negative affect toward depression, and non-medication solutions. Older participants had poorer disease-model attitudes and more negative affect toward depression, whereas younger participants showed poorer help-seeking behavior (*p* < 0.05). The anti-stigma-targeted lecture was superior to the non-targeted lecture in improving disease-model attitudes and non-medication solutions (*p* < 0.05). Multiple regression analyses revealed that each subscale score at post-lecture was strongly dependent on its own baseline subscale score (*p* < 0.01), and that baseline disease-model attitudes also affected post-lecture scores on negative affect toward depression and non-medication solutions (*p* < 0.01).

**Conclusions:**

The educational intervention appears useful for acquiring accurate attitudes toward depression in a medical model. However, other strategies should be considered to enhance help-seeking behavior, especially in younger people.

## Background

The annual number of suicides in Japan exceeded 30,000 between 1998 and 2011, and the suicide rate in Japan ranged from 25.2 to 27.0 per 100,000 people in these years [1]. According to a worldwide survey by the World Health Organization (WHO), the suicide rate in Japan in 2004 was the nine highest in the world [2]. Therefore, there is an urgent need to establish effective strategies for suicide prevention in Japan.

Depression is regarded as one of the major risk factors for suicide in Japan [3] and other countries [4]. However, there still exists stigma surrounding depression among the general population, which may interfere with rational thinking regarding self-management of depression as a medical model approach [5]. The irrational belief that depression can be caused by a nervous/weak personality is relatively strong in Japanese society [6]. Besides, stigmatized individuals tend to hold the belief that suicide can be the ultimate solution to taking responsibility [7]. Such stigmatizing beliefs hinder an individual’s help-seeking from the medical and health professionals who can treat depression [8]. Thus, it has been hypothesized that stigma against depression among the general population might be a potential anti-protective factor for suicide and needs to be corrected as a target for intervention programs. In fact, intervention programs including a focus on destigmatizing depression and motivating help seeking appeared to be practically effective in reducing suicidality according to previous reports in other countries [9, 10]. Therefore, the effects of a stigma-reduction program for depression should be more intensively highlighted and investigated as an initial step for steady suicide prevention in Japan.

Internalized stigma (self-stigma) against mental illness also has a negative impact on self-concept, social relationships, and psychological well-being, which may impede the recovery process from mental illness [11]. People who had experienced involuntary hospitalization by their suicidal behavior reported more internalized stigma than those who did not have such experiences [12]. Besides self-stigma, we should also pay attention to other types of stigma, i.e., personal stigma (one’s personal attitudes toward people with mental illness) [13, 14], perceived stigma (one’s beliefs about social attitudes toward people with mental illness) [13, 14] and public stigma (social prejudice and discrimination against mental illness) [15]. The present study mainly dealt with personal stigma. Previous studies [13, 14, 16–20] assessed personal stigma against the psychiatric services, prevention, human rights, disease, treatment, and help-seeking behaviors. Meanwhile, we adopted a new original scale including the realities of pharmacotherapy of depression and approaches to depressed person in order to facilitate more comprehensive assessment. Furthermore, it is noted that the educational intervention used in the present study consisted of both contextual and biomedical model approaches for depression although previous studies separately adopted either contextual or biological intervention in relation to stigma-reduction approaches [21, 22]. Therefore, the use of combined intervention (contextual plus biological), which may have more potent effects on stigma-reduction, represented a key characteristic of the present study.

Meanwhile, depressive disorder is often accompanied by lack of insight into the disease [23]. Nevertheless, people with depression still tend to seek more help from family or friends than those with alcohol abuse and schizophrenia [24]. Accordingly, family members and intimate friends surrounding depressed individuals can play an important role in identifying possible depressive disorders and helping the sufferer’s access to medical professionals. Therefore, an educational approach that enhances right recognition of depression, and eliminates stigma against depression appears important, not only for high-risk people but also for potential gatekeepers living in the local community. In fact, a recent study demonstrated that a 2-year community-based intervention including cooperation with non-medical community facilitators such as teachers, priests and local media workers resulted in a 24% reduction in frequency of suicidal acts (completed suicides plus suicide attempts) in the city of Nüremberg [9].

These current situations prompted us to develop an efficient intervention for the general population to increase their awareness of depression and decrease stigma against depression. First of all, we investigated baseline levels of public awareness and attitudes toward specifically focused on depression and its treatment, and analyzed the factor structure of them together with the influences of age and gender. Subsequently, we conducted two types of educational interventions (anti-stigma-targeted lecture versus non-targeted general lecture), and examined the results via our original questionnaire to clarify differences in the outcomes between the two lectures.

## Methods

### Interventions

Two types of interventions that were designed as anti-stigma-targeted and non-targeted lectures were separately conducted during the period between January 2009 and December 2010 to compare the differences in their effects. Recruitment of participants was conducted through an advertisement in the newspaper and flyers regarding educational lectures on depression. Both of them were similarly titled “an educational lecture for depression and its treatment” with similar contents of advertisement. These lectures were conducted in the main land of Okinawa prefecture (an isolated island in southern part of Japan) to give scope to acquire knowledge of depression and encourage help-seeking behavior among public citizens. The lecturer gave two types of lectures with different content of power point slides as described below, both of which were given to the audience as face-to-face lectures.

Participants voluntarily attended an anti-stigma-targeted lecture or a non-targeted lecture. The former lecture took 60 minutes and focused on improvement in awareness of and attitudes toward depression and attenuation of anxiety related to its treatments by encouraging help-seeking from others including medical professionals without hesitation or guilt feelings, together with information on the nature of depressive disorders (loss of self-awareness or self-control with regard to the disease), the reality of its treatment course (the benefits of medication combined with psychotherapy and recovery processes), and preferable attitudes for surrounding gatekeepers. The contents were almost identical with those used in a previous study [25].

The other, non-targeted, general lecture also took the same time (60 minutes) although it was not anti-stigma-oriented. The lecture mainly covered comprehensive knowledge of depression (epidemiology, biological etiology, psychological aspects, associated life events, symptoms, suicidal risk, and clinical course including remission and relapse) and provided an outline of its treatments (standard pharmacotherapy and psychotherapy), which was only designed for public citizens to get correct knowledge of depression and its treatments.

### Participants

Participants were not randomly assigned to either of the two lectures (an anti-stigma-targeted or a non-targeted lecture) due to our naturalistic study design. Although group allocation was not randomly assigned, participants were recruited by similar advertising methods during different periods.

Eight-hundred and twenty-seven participants received the anti-stigma-targeted lecture. Among them, 688 participants (221 males and 467 females) gave full cooperation with the questionnaires as voluntary respondents. Finally, we extracted 467 questionnaires that had no omission of data (160 males and 307 females; 40.4 ± 13.6 years).

Meanwhile, 598 participants attended the non-targeted educational lecture, and 532 participants (114 males and 418 females) were voluntary respondents to the questionnaires. Among them, 367 questionnaires had no omission data (85 males and 282 females; 39.1 ± 12.4 years).

Thus, baseline awareness and attitudes were analyzed by using the 834 participants in total (245 males and 589 females; 39.8 ± 13.1 years) with complete data. The overall recovery rate for respondents who provided completed data was 834/1425 (58.5%). The distribution for age was as follows: 150 (≦29 years), 165 (30 -39 years), 178 (40 -49 years), 234 (50 -59 years) and 107 participants (≧60 years).

### Measures

The 18-item questionnaire on depression in Japanese (Table 1), which we originally developed and used in a previous study [25], was administered to each participant. The questionnaire was completed before and immediately after the lectures. Since the factor structure of this 18-item questionnaire had not been validated in our previous study due to specific subjects (university students) [25], we aimed to test its reliability and validity among general population in the present study.

**Table 1:** The 18-item questionnaire for attitudes toward depression and its treatments

うつ病についてどう思いますか	Attitudes toward Depression
1. 恐い病気である	1. Fear : Depression is fearful disease
2. うつ病についてあまり知らない	2. Lack of knowledge : I do not have enough knowledge of depression
3. 性格的に弱い人がうつ病になる	3. Weakness : Weak people suffer from depression
4. 自分がうつ病になったら恥ずかしい	4. Shame : Suffering from depression is shameful
5. うつ病になると周りに迷惑を掛ける	5. Burden to others : Suffering from depression may bother others
6. うつ病の人は苦しい現実から逃げている	6. Escaping from reality : Depression is an escape from reality
7. 自分はうつ病にかかった時に自覚できる	7. Overconfidence in self-awareness : I can be fully aware of my depressive state
8. うつ病は気の持ちようで克服できる	8. Self-manageable disease : I can get over depression by myself
うつ病になったらどうしますか	Attitudes toward treatments
1. ためらわずに誰かに助けを求めますか?	1. Help-seeking :Do you ask for someone’s help without hesitation?
2. 家族に相談しますか?	2. Consulting with family :Do you consult with your family?
3. かかりつけ医を受診しますか?	3. Visiting general practitioners : Do you go to see a general practitioner?
4. 精神科を受診しますか?	4. Visiting Psychiatrists :Do you go to see a psychiatrist?
5. できればカウンセリングだけで治したいですか?	5. Over-expectation from counseling Will you get over depression only by counseling?
6. 抗うつ薬はなるべく服用したくないですか?	6. Reluctance to medication : Are you reluctant to take antidepressants?
7. 薬がやめられないのではと心配になりますか?	7. Concern for drug dependence : Are you afraid of drug dependence?
8. すぐによくならないと薬を止めてしまいますか?	8. Adherence to acute medication : Can you wait for slow-onset of drug effects?
9. よくなったら、すぐに抗うつ薬を止めますか?	9. Necessity of maintenance therapy : Will you stop medication soon after getting well?
10.落ち込んでいる人にはまず励ましますか?	10. Approaches to depressed others : Do you believe that encouragement will help a depressed person?

The questionnaire contains 8 items to assess common misconceptions surrounding depression (fear, lack of knowledge, weakness, shame, burden to others, escaping from reality, overconfidence in self-awareness, and self-manageable disease), and 10 items assessing attitudes toward its treatments (help-seeking, consulting with family, visiting general practitioners, visiting psychiatrists, over-expectations from counseling, reluctance to medication, concern for drug dependence, adherence to acute medication, necessity of maintenance therapy, and approaches to depressed others). The two domains (attitudes toward depression and attitudes toward treatments) were just presented as convenient subcategories for our questionnaire to respondents.

Personal weakness as a cause of depression, overconfidence in self-awareness of depression, depression as a self-manageable disease, hesitation to seek help from family, general practitioners and psychiatrists, reluctance to medication in contrast to over-expectations from counseling and over-concern for drug dependence have been already identified as common stigmas related to depression in previous studies [6, 13, 16, 19, 20]. Other possible items related to misconceptions regarding depression were developed according to the researchers’ clinical experience with depression.

Attitudes were evaluated on a 5-point Likert scale (1 = *very negative* to 5 = *very positive*). In principle, the effects of the two lectures as interventions were assessed by using a pretest-posttest design. Higher scores indicate a better understanding of depression.

### Data analysis

After anonymous questionnaires were collected from participants, the data was statistically analyzed as grouped data. The study protocol was approved by the Ethics Committee of the University of the Ryukyus.

A chi-square test and residual analysis were used to investigate categorical variables for background data on the participants when comparing the groups receiving the anti-stigma-targeted lecture and the non-targeted lecture. Factor analysis of baseline scores after Promax rotation was conducted on the 18 questionnaire items. Student’s *t*-test was used for analysis of the gender effect of baseline and post-lecture. A two-way analysis of variance (ANOVA) was used for comparison among the 5 age groups and comparison of the effect of the anti-stigma-targeted lecture with that of the non-targeted lecture. Multiple regression analysis was applied to examine the determinants of improved attitudes after intervention. Independent variables were the background of the participants (gender and age), type of intervention (non-targeted/anti-stigma-targeted lecture), and baseline attitudes (disease-model attitudes, help-seeking behavior, negative affect toward depression, and non-medication solutions) in these models.

A two-tailed *p*-value less than 0.05 was regarded as statistically significant. IBM SPSS statistics 22 and Stat vies 5.0 were used for these statistical analyses.

## Results

### Background analyses of the two intervention groups

The backgrounds of the participants in the anti-stigma-targeted and non-targeted lectures were compared. A chi-square test and residual analysis showed that the proportion of females was higher in the group receiving the non-targeted lecture than those receiving the anti-stigma-targeted lecture (76.8% versus 65.7%; χ^2^ = 837.60, df = 4, *p* < 0.01). The proportion of participants over 60 years of age was higher in the anti-stigma-targeted lecture group (8.7% vs. 16.1%; χ^2^ = 12.17, df = 4, *p <* 0.05). Student’s *t-*test was performed to compare demographics between respondents who gave completed data and those who were excluded from the study due to incomplete data. The mean age was younger in respondents with complete data than that in those with incomplete data within each lecture group (anti-stigma-targeted lecture group: 40.4 ± 13.6 versus 45.8 ± 14.1 ; non-targeted lecture group: 39.1 ± 12.4 versus 45.4 ± 12.1).

### Factor analysis

Factor analysis of baseline scores after Promax rotation of 834 participants for the 18 items on attitudes toward depression and its treatment are summarized in Table 2. Four distinct factors were extracted that constituted four subscales: disease-model attitudes (approaches to depressed others, self-manageable disease, adherence to acute medication, necessity of maintenance therapy, lack of knowledge, and escaping from reality; Cronbach’s α = 0.73); help-seeking behavior (visiting general practitioners, consulting with family, visiting psychiatrists, and help-seeking; Cronbach’s α = 0.73); negative affect toward depression (burden to others, shame, fear, and weakness; Cronbach’s α = 0.66); and non-medication solutions (reluctance to medication, over-expectation from counseling, and concern for drug dependence; Cronbach’s α = 0.75). The item overconfidence in self-awareness was excluded from factor analysis due to its very low factor loading.

**Table 2:** Factor analysis of baseline scores in the 18 items for attitudes toward depression and its treatments (*N* = 834)

	Disease-model attitudes (Cronbach’s α = 0.73)	Help-seeking behavior (Cronbach’s α = 0.73)	Negative affect toward depression (Cronbach’s α = 0.66)	Non-medication solutions (Cronbach’s α = 0.75)
Approaches to depressed others	0.69			
Self-manageable disease	0.65			
Adherence to acute medication	0.62			
Necessity of maintenance therapy	0.54			
Lack of knowledge	0.38			
Escaping from reality	0.36			
Visiting general practitioners		0.93		
Consulting with family		0.56		
Visiting psychiatrists		0.52		
Help-seeking		0.47		
Burden to others			0.71	
Shame			0.60	
Fear			0.57	
Weakness			0.41	
Reluctance to medication				0.95
Over-expectation from counseling				0.47
Concern for drug dependence				0.41
Overconfidence in self-awareness		(-0.13)		

### Gender and age effects

Gender and age effects of baseline and post-lecture scores are summarized in Table 3. Student’s *t-*test showed that males had a lower post-lecture score on the subscale of negative affect toward depression (t (832) = 2.3, *p* < 0.05). A two-way ANOVA showed that at both baseline and post-lecture, the older age groups (50 -59 and ≧ 60) had lower subscale scores for disease-model attitudes and/or negative affect toward depression, whereas the younger age groups (≦29 and 30 -39 years) showed poorer help-seeking behavior (*p* < 0.05).

**Table 3:** Gender and age effects of baseline and post-lecture (*N* = 834)

	Baseline				Post-lecture			
	Disease-model attitudes	Help-seeking behavior	Negative affect toward depression	Non-medication solutions	Disease-model attitudes	Help-seeking behavior	Negative affect toward depression	Non-medication solutions
Gender								
Males (*n* = 245)	19.2 ± 5.1	13.0 ± 4.1	9.8 ± 3.2	7.6 ± 3.2	22.6 ± 4.5	14.7 ± 3.4	12.1 ± 3.4 *	9.5 ± 3.1
Females (*n* = 589)	19.5 ± 4.9	13.5 ± 3.6	10.1 ± 3.1	7.3 ± 3.1	22.7 ± 4.7	15.1 ± 3.4	12.7 ± 3.5	9.6 ± 3.3
Age								
≦29 (*n* = 150)	20.0 ± 4.4	12.0 ± 3.3^cde^	10.4 ± 3.0	7.4 ± 2.7	23.6 ± 3.7	14.3 ± 3.2^cd^	12.6 ± 3.0	9.7 ± 2.8
30-39 (*n* = 165)	20.3 ± 4.9	12.8 ± 3.9^e^	10.0 ± 3.0	7.6 ± 3.1	23.3 ± 4.6	14.7 ± 3.5	12.5 ± 3.2	9.4 ± 3.1
40-49 (*n* = 178)	20.1 ± 4.7	1 3.6 ± 3.7	10.3 ± 3.0	7.6 ± 3.3	23.2 ± 4.2	15.4 ± 3.4	13.2 ± 3.3	9.8 ± 3.2
50-59 (*n* = 234)	18.9 ± 5.0^b^	13.8 ± 3.7	10.0 ± 3.4	7.2 ± 3.3	22.0 ± 5.0^a^	15.3 ± 3.5	12.5 ± 3.8	9.6 ± 3.4
≧60 (*n* = 107)	17.1 ± 5.0^abcd^	14.4 ± 3.7	9.0 ± 3.1^ac^	7.1 ± 3.0	20.8 ± 5.1^abc^	15.3 ± 3.2	11.5 ± 3.6^c^	9.3 ± 3.5

* Significance from females (*p* < 0.05)

^a-e^: Significance from the age groups of ≦ 29^a^, 30-39^b^, 40-49^c^, 50-59^d^ and ≧ 60^e^ years (*p* < 0.05)

### Effects of different interventions

The effects of the different interventions based on baseline and post-lecture scores are summarized in Table 4. A two-way ANOVA showed that all post-lecture scores improved for both lectures (*p* < 0.05). The anti-stigma-targeted lecture was superior to the non-targeted lecture in improving disease-model attitudes and non-medication solutions (*p* < 0.05).

**Table 4:** Comparison of intervention effects between the anti-stigma-targeted and non-targeted lectures (*N* = 834)

	Disease-model attitudes	Help-seeking behavior	Negative affect toward depression	Non-medication solutions
Non-targeted (*n* = 367)				
Baseline	19.2 ± 4.9	13.5 ± 3.6	10.2 ± 3.1	7.5 ± 3.2
Post-lecture	21.3 ± 4.7^a^	14.9 ± 3.4^a^	12.4 ± 3.4^a^	9.1 ± 3.2^a^
Targeted (*n* = 467)				
Baseline	19.6 ± 5.0	13.2 ± 3.8	9.9 ± 3.2	7.3 ± 3.1
Post-lecture	23.7 ± 4.3^ab^	15.1 ± 3.4^a^	12.7 ± 3.5^a^	9.9 ± 3.2^ab^

a : Significance from baseline (*p* < 0.05)

b Significance from non-targeted lecture (*p* < 0.05)

### Determinants of post-intervention effects

Possible determinants of post-intervention effects were examined and are summarized in Table 5. Multiple regression analyses revealed that each subscale score at post-lecture was strongly dependent on its own baseline subscale score (*p* < 0.01), and that baseline disease-model attitudes also affected post-lecture scores on two other subscales: negative affect toward depression and non-medication solutions (*p* < 0.01). The anti-stigma-targeted lecture significantly contributed to greater improvement in subscale scores for disease-model attitudes (*p* < 0.01), negative affect toward depression (*p* < 0.05), and non-medication solutions (*p* < 0.01). Older age was associated with poorer disease-model attitudes (*p* < 0.01), while gender did not affect any post-lecture scores.

**Table 5:** Multiple regression analyses of possible determinants for the 4 subscale scores of post-lecture (*N* = 834)

	Disease-model attitudes		Help-seeking behavior		Negative affect toward depression		Non-medication solutions	
	β	*P*	β	*P*	β	*P*	β	*P*
Gender	0.020	0.39	0.026	0.30	0.052	0.57	0.050	0.07
Age	-0.072	0.00	-0.008	0.76	-0.003	0.93	0.023	0.42
Lecture (non-targeted/anti-stigma-targeted lecture)	0.238	0.00	0.051	0.05	0.068	0.01	0.139	0.00
Baseline scores								
Disease-model attitudes	0.671	0.00	0.059	0.08	0.129	0.00	0.126	0.00
Help-seeking behaviors	-0.025	0.37	0.647	0.00	0.046	0.15	-0.008	0.81
Negative affect toward depression	0.022	0.43	0.007	0.82	0.545	0.00	0.057	0.08
Non-medication solutions	0.008	0.78	0.053	0.10	-0.029	0.41	0.514	0.00
	*R* = 0.74	*R* ^2^ = 0.55	*R* = 0.70	*R* ^2^ = 0.48	*R* = 0.63	*R* ^2^ = 0.39	*R* = 0.63	*R* ^2^ = 0.40
	*F* = 146.5	*p* = 0.000	*F* = 111.0	*p* = 0.000	*F* = 77.7	*p* = 0.000	*F* = 78.7	*p* = 0.000

β: standardized partial regression coefficient. Dummy variables were used for gender (male: 0, female: 1) and types of the lectures (non-targeted: 0, anti-stigma-targeted: 1)

## Discussion

Although personal stigma and social distance about depression and suicidality are relatively greater in Japan than in other countries, few studies have been conducted that used an intervention program to reduce stigma against mental illness [14, 26]. In fact, public awareness campaigns about depression and suicide can actually lead to modest improvement in public knowledge of and attitudes toward depression and suicide [27]. Given such interventional effects can differ among various approaches that differ in focus and quality, we conducted two different types of interventions to improve awareness and attitudes for the general public regarding depression, and compared the effects of an anti-stigma-targeted lecture with that of a non-focused general lecture regarding depression. As a first step, the focused outcome in the present study was immediate changes in attitudes after the intervention rather than durable effects on suicidality in the community.

Four main results are summarized as follows: 1) Public attitudes toward depression consisted of 4 distinct elements, which were disease-model attitudes, help-seeking behavior, negative affect toward depression, and non-medication solutions; 2) Older participants had poorer disease-model attitudes and more negative affect toward depression, whereas younger participants showed poorer help-seeking behavior; 3) The anti-stigma-targeted lecture was superior to the non-targeted lecture in improving disease-model attitudes and non-medication solutions; 4) Final attitudes were strongly dependent on their baseline levels and affected by baseline disease-model attitudes.

Gender differences were only minimally observed as more negative affect in males even after the intervention. This finding may be explained by a tendency for males, as compared females, to be more controlled by inner shame and guilt feelings and have more persistent misbelief that depression comes from a weakness in personality. Therefore, it should be warned of that denial of depression can be a problem in males due to their less correctable negative affect toward depression.

Generation effects were more apparent in the present study. Poor disease-model attitudes and negative affect toward depression in the elderly are in line with a previous suggestion that Japanese elderly people tend to underestimate depression and often mix up their depressed state with the aging process, which may unfortunately lead to delays in seeking help from mental health professionals [28]. Therefore, it is important for the elderly to receive educational programs regarding how to recognize the bright side of medical treatments, such as viewing depression as a treatable disease, and lower their threshold to seek medical support [29]. Meanwhile, less seeking of medical help in younger generations can be partly explained by the fact that younger people prefer to seek support from surrounding people rather than unfamiliar medical professionals [30]. Additionally, since younger people may seek the first-touch help from their friends rather than their family, an intervention enhancing peer-support and self-help capability as gatekeepers is a more important strategy for younger people. It is also suggested that an Internet-based intervention can be helpful in reducing depression and anxiety [31, 32], which may become a more accessible and preferable tool than face-to-face consultations for younger people to encourage their help-seeking action.

The anti-stigma-targeted lecture apparently had greater enhancing effects on attitudes toward depression as a medical model, which could be followed by attenuated public orientation toward non-medication solutions. The Defeat Depression Campaigns using mass media (newspaper/magazine articles, radio/television programs, and other media activities) in the United Kingdom finally failed to improve public attitudes toward medical treatments of depression [16]. Thus, the face-to-face intervention with the clear intention of anti-stigmatization may have a stronger impact on positive changes in attitudes toward medical treatments of depression than a wide but superficial campaign with general information through mass media. Meanwhile, we must admit that a lecture-based approach may be limited in terms of population reach and its impact on help-seeking behavior, because even the anti-stigma-targeted lecture promoted only marginal improvement in the present study. In order to encourage help-seeking action, other strategies that are emotionally moving, motivation-enhancing, and easily accessed approaches, such as the use of a short promotion movie (successful help-seeking), simulative peer-supporting experiences by role play, and Internet-based support from mental health professionals may be needed as forwarded strategies.

Systematic gatekeeper training has been regarded as one of the promising strategies to identify high risk individuals and connect them with medical professionals in the community [9]. It is also suggested that education of gatekeeper candidates may be more effective and efficient among people with less stigma at baseline, especially those with better disease-model attitudes, given the present study clearly indicated that post-education levels of anti-stigmatization were mainly determined by baseline attitudes toward depression, and that disease model recognition was the most influential in the overall anti-stigmatization process.

Although the educational intervention might have had less durable effects, it is also true that even one lecture efficiently showed a considerable impact to alter the awareness and images of depression and its treatments in public citizens. Thus, continuous efforts to develop educational interventions for public citizens to accept treatment of depression as a medical model, simultaneously with refining of the approaches by repeated feedback from outcome research, will remain a timeless and meaningful strategy as a basic step in suicide prevention.

The present study has several limitations. First, we should consider the bias of motivation and characteristics of the participants. The present findings may not be generalizable to the overall population due to the lecture groups not being well- balanced in terms of age and gender distributions. Second, we adopted the original but non-validated questionnaire for assessment, although its validation formed one of the express purposes of the present study. Third, there is a major methodological limitation due to its naturalistic study design, because participants were not randomly allocated to either the anti-stigma-targeted or the non-targeted lecture. In addition, no information on educational status or health literacy seems to be a methodological limitation, which may have affected the results. Moreover, the possibility that exclusion of subjects with incomplete data (older than those with complete data) may have caused age bias cannot be entirely ruled out. Fourth, long-term durable effects were not examined because only the immediate effects of the intervention were studied. A follow-up study would be needed examining attitudes over a specified period of time. Fifth, interventional effects should be evaluated by behavioral outcomes such as an increased consultation rate for psychiatric services or decreased suicide deaths. To clarify these issues, further follow-up studies will be needed.

## Conclusions

In conclusion, the present study suggests that the main target of a lecture-based educational intervention for public citizens is the acquisition of right awareness of and correct attitudes toward depression and its treatments in a medical model, which can secondarily attenuate negative affect toward depression and medication. However, other strategies should be considered for enhancing help-seeking behavior, especially in younger people.

## Abbreviations

ANOVA: Analysis of variance
WHO: World Health Organization
